# Outcomes of manually modified microvascular plugs to pulmonary flow restrictors in various congenital heart lesions

**DOI:** 10.3389/fcvm.2023.1150579

**Published:** 2023-07-10

**Authors:** Raymond N. Haddad, Jamie Bentham, Ahmed Adel Hassan, Mahmoud Al Soufi, Osama Jaber, Issam El Rassi, Mohamed Kasem

**Affiliations:** ^1^ M3C-Necker, Hôpital Universitaire Necker-Enfants Malades, Assistance Publique-Hôpitaux de Paris (AP-HP), Paris, France; ^2^ Department of Congenital Cardiology, Leeds Teaching Hospitals NHS Trust, Leeds, United Kingdom; ^3^ Department of Pediatric Cardiology, Heart Centre of Excellence, Al Jalila Children’s Speciality Hospital, Dubai, United Arab Emirates; ^4^ Department of Congenital Cardiac Surgery, Leeds Teaching Hospitals NHS Trust, Leeds, United Kingdom; ^5^ Department of Pediatric Cardiac Surgery, Heart Centre of Excellence, Al Jalila Children’s Speciality Hospital, Dubai, United Arab Emirates

**Keywords:** congenital heart disease, microvascular plug, pulmonary artery band, pulmonary flow restrictor, transcatheter intervention

## Abstract

**Background:**

The development of microvascular plugs (MVPs) has enabled novel transcatheter deliverable endoluminal pulmonary flow restrictors (PFRs) with the potential to treat newborns and infants with life-threatening congenital heart diseases (CHDs) in a minimally invasive manner. We present our experience to evaluate the efficacy of this concept in controlling pulmonary blood flow in various CHDs.

**Methods:**

Retrospective clinical data review of patients with CHD and pulmonary over-circulation who received bilateral PFRs percutaneously.

**Results:**

Twenty-eight PFRs (7 MVP-5Q, 12 MVP-7Q, and 9 MVP-9Q) were finally implanted in 14 patients with a median age of 1.6 months (IQR, 0.9–2.3) and a median weight of 3.1 Kg (IQR, 2.7–3.6). Nine patients had large intra-cardiac left-to-right shunts (including 3 with fatal trisomy and palliative programs), 2 had borderline left ventricles, 2 had Taussig-Bing anomaly, and one had a hypoplastic left heart. Four patients had concomitant ductal stenting. Two MVP-5Qs were snare-removed and upsized to MVP-7Q. Patients experienced a significant drop in oxygen saturation and Qp/Qs. All patients were discharged from the ICU after a median of 3.5 days (IQR, 2–5.8) postoperative. Five patients had routine inter-stage catheterization and no device embolization or pulmonary branch distortion was seen. Fourteen (50%) PFRs were surgically explanted uneventfully on a median of 4.3 months (IQR, 1.2–6) post-implantation during biventricular repair in 6 patients and stage-2 palliation in one patient. The latter died 1 month post-operative from severe sepsis. Four patients are scheduled for surgical PFR removal and biventricular repair. Two patients with trisomy 18 died at 1 and 6.8 months post-procedure from non-cardiac causes. One patient with trisomy 13 is alive at 2.7 months post-procedure.

**Conclusion:**

It is feasible to bespoke MVPs and implant them as effective PFRs in various CHDs. This approach enables staged left ventricular recruitment, comprehensive stage-2 or biventricular repair with lower risk by postponing surgeries to later infancy. Device explantation is uneventful, and the outcomes afterward are promising.

## 1. Introduction

Control of distal pulmonary artery (PA) pressure and blood flow is a critical step in palliating babies with complex congenital heart disease (CHD). A novel, less invasive, and less aggressive potential alternative is to manually convert the thin polytetrafluoroethylene (PTFE)-covered, nitinol-framed, self-expandable microvascular plugs (MVPs) (Medtronic Inc., USA) from occlusion devices into endovascular pulmonary flow regulators (PFRs) (1–3). Following the animal work done by Khan et al. (1), the initial human experience came from Giessen, Germany where they used this technique for non-surgical transcatheter stage-1 in six newborns with hypoplastic left heart syndrome (HLHS) and variants, and the outcomes were excellent (2, 3). The same technique was also applied by an American group who reported their experience in a small series of six patients as a word of caution before any widespread application of this technique (4). In this study, we expand upon earlier findings to describe and evaluate our experience in using this novel technique for patients with different congenital lesions in which balanced pulmonary and systemic circulation was needed.

## 2. Patients and methods

### 2.1. Study design

We performed a retrospective clinical data review of all patients with CHDs who had transcatheter implantation of manually modified MVPs to PFRs at our institutions between September 2021 and September 2022. Standard safety and outcomes were assessed. All cases were discussed and approved during multi-disciplinary team meetings before the intervention. Approval from the institutional review board was obtained. Signed informed consent was obtained for the patient's legal guardians. This approach was applied as a part of total percutaneous stage 1 palliation in patients with a single ventricle. The PFRs were also used as short-term palliation in patients with two ventricles and significant left-to-right shunts such as atrioventricular septal defect (AVSD) or large ventricular septal defect (VSD) before complete repair. Patients with fatal trisomy 13 or 18 had palliative care programs. Some patients were deemed high-risk surgical candidates or needed time for staged left ventricular recruitment.

### 2.2. MVP device and delivery system

**Figure 1:**
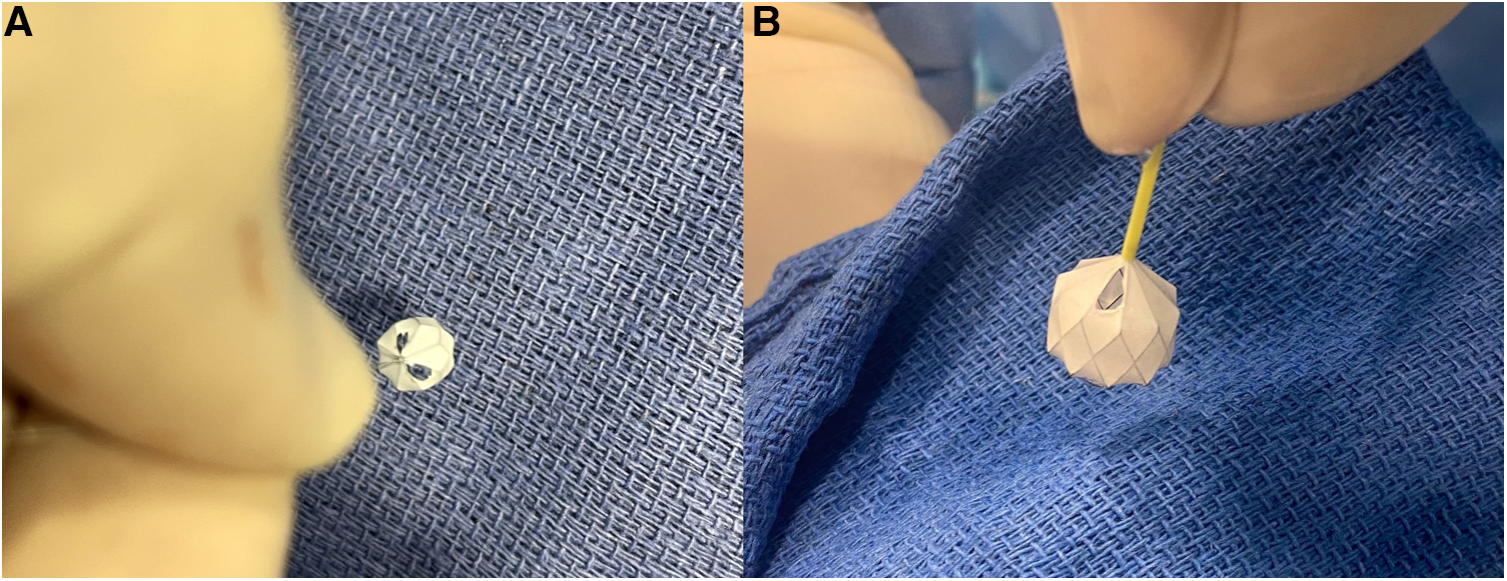
Manually-modified MVP-5Q after removal of the PTFE membrane within two opposing triangles (**A**). Manually-modified MVP-7Q with one fenestrated triangle at the most proximal inflow V-line of the nitinol wire (**B**).

The MVP is a US FDA-approved and CE-marked self-expanding mechanical occlusion device. It is designed as a single-cage hexagonal framework made of a flexible, laser-cut Nitinol wire (Figure 1). The main body consists of an ovoid-shaped cylinder with both extremities tapering down to the center of the axis. The PTFE-coating has an asymmetrical parachute design, starting from the proximal tapering to the end of the tubular part whereas the distal tapering remains a bare segment. There is a single radiopaque platinum marker at each end. The plug is packaged soldered to a 180 or 165 cm-long highly flexible delivery wire. The detachment is mechanical with anticlockwise torque. A 4 cm plastic sleeve is present over the delivery cable to facilitate device loading. The device is currently available in four sizes with unconstricted diameters of 5.3, 6.5, 9.2, and 13 mm. The MVP-3Q and MVP-5Q consist of 6 and 8 covered segments, respectively. The MVP-7Q and MVP-9Q both consist of 10 covered segments. The technical specifications of the MVP are outlined in Table 1.

**Table 1:** Technical specifications of the 4 MVP models.

MVP Models	Recommended vessel size	Compatible catheter	Unconstrained length	Unconstrained outer diameter	Unconstrained device circumference	Unconstrained device area	Number of covered segments	Base segment area	Central angle of base segment	Base segment cord length
MVP-5Q	3.0–5.0 mm	0.027 in	12 mm	6.5 mm	20.42 mm	33.18 mm^2^	8	4.15 mm^2^	45°	2.49 mm
MVP-7Q	5.0–7.0 mm	0.041 in (4-Fr)	16 mm	9.2 mm	28.9 mm	66.48 mm^2^	10	6.65 mm^2^	36°	2.84 mm
MVP-9Q	7.0–9.0 mm	0.043 in (5-Fr)	18 mm	13.0 mm	40.84 mm	132.73 mm^2^	10	13.27 mm^2^	36°	4.02 mm

### 2.3. Device selection protocol

Considering that MVPs are originally designed for occlusion of less dynamic peripheral vasculature, the sizes of the manually modified devices were oversized according to the branch PA diameter. We implanted MVP-5Q for vessels with a diameter up to 4 mm, MVP-7Q for up to 6 mm, and MVP-9Q for up to 8 mm.

### 2.4. Adjusted modification technique of MVP device

The first operator stabilized the position of the MVP by holding the delivery cable with one hand and fixing the distal radiopaque marker with the other hand with toothless forceps. It is important not to hold the nitinol cage because it is flimsy. The second operator used a thin carbon steel surgical scalpel blade No. 11 (Swann-Morton®, England) to slice the PTFE membrane within the selected diamond at the most proximal inflow V-line of the nitinol wire. We speculated that this will keep the fenestration open even if there is compression on the device after implantation. The PTFE membrane was held under slight tension with toothless forceps. The distal base of the triangle was sliced with the scalpel to fenestrate half a diamond (i.e., one triangle) (Figure 1). The PTFE was cut from the other nitinol V-line of the diamond when a fenestration within an entire diamond was decided. In patients with a single ventricle program and patients with two ventricles and excessive pulmonary blood flow, we went as tight as possible to aggressively lower the PA pressure and tightly regulate the pulmonary blood flow. Therefore, we fenestrated 2 triangles in 2 different opposing diamonds on the MVP-5Q (Figure 1A) and one triangle on the MVP-7Q (Figure 1B) and MVP-9Q. We fenestrated an entire diamond in patients with failure of previous surgical PA band or with Taussig-Bing anomaly where the drop in pulmonary-systemic flow ratio (Qp/Qs) mismatch is needed without significant cyanosis. The *ex-vivo* theoretical calculated area of the fenestrated triangle within each model and the equivalent inner diameter of a circle with the same area are outlined in Table 2. The MVP-3Q is too small and was not used in this approach.

**Table 2:** *Ex-vivo* theoretical calculated area of the fenestrated triangle within each model and the equivalent inner diameter of a circle with the same area.

MVP Models	Base Triangle Fenestration				Equivalent inner diameter of a circle with the same area
	Side a length	Side b length	Side c length	Area^a^	
MVP-5Q	3.25 mm	3.25 mm	2.49 mm	3.74 mm^2^	2.2 mm
MVP-7Q	4.6 mm	4.6 mm	2.84 mm	6.21 mm^2^	2.8 mm
MVP-9Q	6.5 mm	6.5 mm	4.02 mm	12.43 mm^2^	3.9 mm

^a^ Calculated using Heron's Formula.

### 2.5. Interventional procedure

The intervention was performed under general anesthesia, antibiotics prophylaxis, systemic heparinization, and biplane fluoroscopy. Anemia was corrected before the procedure to avoid jeopardizing the hemodynamic assessment. Femoral venous access was obtained with a 4 or 5-Fr Prelude sheath introducer (Merit Medical, USA). 6-Fr venous access was obtained in case of concomitant ductal stenting or balloon atrial septostomy. Jugular access was used in case of compromised femoral access. A 4-Fr arterial line was obtained. The QP/QS measurement was performed under an inspired fraction of oxygen (FiO2) at 21%. The tricuspid valve was crossed with a standard 2.7-Fr Progreat (Terumo Corp., Japan) or a steerable SwiftNINJA® (Merit Medical Systems, Inc., USA) microcatheters. A 4-Fr multipurpose Radiofocus GlideCath (Terumo Corp., Japan) was used for selective hand angiograms in the left pulmonary artery (LPA) in 30° left anterior oblique/lateral 90° projections and in the right pulmonary artery (RPA) in 30° right anterior oblique/lateral 90° projections (Supplementary Video S1). Angiographic measurements of proximal and distal diameters of both branch PAs were taken and compared to the measurements on transthoracic echocardiography (TTE).

When needed, balloon atrial septostomy was done before bilateral PFR placement and finally, the ductal stenting, as is routine in the Giessen hybrid approach (5). It is trickier to get to the LPA after ductal stenting and the left PFR can serve as a useful landmark of the pulmonary end of the arterial duct. The LPA was always dealt with first because it is easier to access the RPA if the LPA is partially plugged. The distal LPA was accessed with a combination system of standard or steerable microcatheter and a 0.014-inch coronary wire. The glide catheter was railed over the telescoping system and placed distally to the landing zone (Supplementary Video S2). A Y-connector was placed at the end of the catheter to prevent blood loss and allow angiograms.

**Figure 2:**
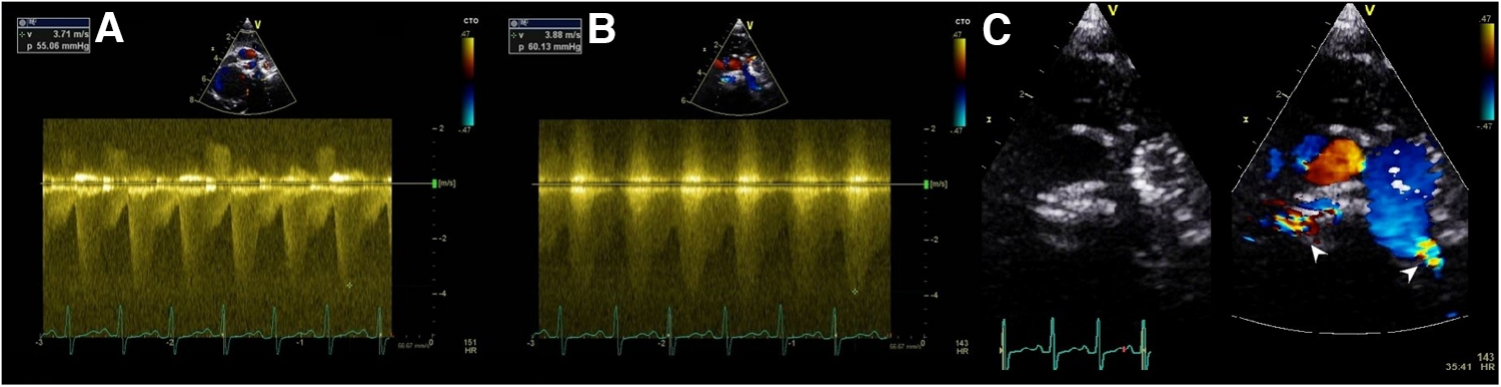
Ultrasound Doppler tracing showing a systolic-diastolic flow profile of effective PFRs in LPA (**A**) and RPA (**B**) of patient no.5. 2D and color-Doppler ultrasound short-axis view of PFRs in both PA branches (white pointed arrows) (**C**).

After size selection, preparation, and modification of the MVP device, the system was then inserted in the same glide catheter through the Y-connector. It is important to go distally, place the distal radiopaque marker at the target area, and then rapidly uncover the MVP (Supplementary Video S3). The distal part of the MVP is bare-metal, thereby placing the distal marker at the take-off point of the first upper lobe branch PA will not jail it. Pulling back the deployed device, if not satisfied with the position, was avoided because it will likely cause proximal migration of the MVP into the MPA. Before release, a hand angiogram was done to check the patency of the upper lobe branches and the relationship of the MVP's proximal end with the pulmonary valve. A 2D short-axis ultrasound was done to confirm the device position and to measure the pressure gradient with continuous-wave Doppler tracing (Figure 2).

The RPA was then engaged using the microcatheter, but more carefully not to accidentally cross to LPA, even with wire, as this could change the geometry of the left PFR. The coronary wire was then placed distally over which the glide catheter was taken distally in the RPA. After the right PFR deployment, we did an exit angiogram (Supplementary Videos S4, S5) and another set of invasive pressure and oxygen saturation measurements. The goal of the procedure is to drop the oxygen saturation on the arterial blood gas by 10% and reduce the QP/QS mismatch by 50%. In patients with left-to-right shunt defects, the goal was also to obtain a minimal peak Doppler gradient of 40 mmHg on per-procedural ultrasound assessment.

### 2.6. Follow-up protocol

Post-procedure, all patients were sent sedated and intubated to the intensive care unit, and were weaned progressively from ventilator overnight or the next day. Follow-up drug treatment consisted of continuous infusion of heparin (20 UI/kg/h) for 2 days, overlapped by daily oral clopidogrel (0.2 mg/kg) and acetylsalicylic acid (5 mg/kg). In all patients receiving diuretics (furosemide 1 mg/kg/dose every 8 or 12 h and spironolactone 1–2 mg/kg daily given in 1–2 divided doses) before the intervention, diuretic therapy was kept identical the next post-procedure day. Patients were slowly weaned off the diuretic therapy over 2–7 days according to the oxygen saturation and ultrasound assessment of the intravascular volume. No other cardiovascular drugs were used. Following discharge, routine outpatient follow-ups were scheduled. The assessment included clinical evaluation, physical examination, saturation measurements, and TTE. Before surgical explantation of PFRs, follow-up cardiac catheterization was performed in patients with borderline anatomy and physiology to decide on the repair project. Patients with large intra-cardiac left-to-right shunts (AVSD and VSD) were directly sent for surgical explantation of PFRs and biventricular repair.

### 2.7. Statistical analyses

Statistical analyses were performed using SPSS, Version 22.0 for Macintosh (IBM, Armonk, NY, USA). Categorical variables were reported as frequency and percentage and continuous variables were represented as median with IQR (interquartile range) as appropriate. Statistical analysis for continuous variables was conducted using Mann–Whitney *U* test. A *p*-value <0.05 was considered statistically significant. All reported *p* values are two-sided.

## 3. Results

During the study period, 28 PFRs (7 MVP-5Qs, 12 MVP-7Qs, and 9 MVP-9Qs) were finally implanted in 14 patients with a median age of 1.6 months (IQR, 0.9–2.3) and weight of 3.1 Kg (IQR, 2.7–3.6). Nine patients had large intra-cardiac left-to-right shunts, 2 had borderline left ventricles, 2 had Taussig-Bing anomaly, and one had a hypoplastic left heart. Six patients with large intra-cardiac left-to-right shunts had trisomy disorders (including 3 with fatal trisomy and palliative programs). The patients' clinical characteristics are outlined in Table 3.

**Table 3:** Patients’ clinical characteristics.

CN	Gender	Age	W/H	CHD	Associated syndrome	Ventilation support	Inotropic support	Baseline Qp/Qs	Baseline Spo2	Repair program	Concomitant percutaneous procedure
1	F	2.4	3.6/50	Right-dominant partially imbalanced AVSD, Aortic CoA, s/p surgical PA band, and Aortic CoA repair	–	I	No	2.2	100	BiV	–
2	F	1	3.9/48	Taussig Bing anomaly, Aortic CoA Coronary artery anomaly Slightly dysplastic PV (Previously treated neonatal infection)^a^	–	NI	No	2.8	93	BiV	Ductal stenting, BAS
3	F	0.4	2.7/47	Subaortic stenosis, Aortic CoA, VSD Borderline LV	–	NI	No	3.2	100	Potential BiV	Ductal stenting
4	F	2	2.7/43	VSD, PDA, ASD	T18	I	No	2.9	99	Palliative	–
5	M	1.2	3.2/48	AS, Aortic CoA, No VSD Borderline LV s/p AS ballooning, BAS (Transfer for another center)^a^	–	I	Yes	1.8	99	Potential BiV	Ductal stenting
6	M	2.9	5/52	VSD, ASD	–	NI	No	3.5	100	BiV	–
7	M	2.4	3.6/53	Complete balanced AVSD	–	NI	No	3.2	100	BiV	–
8	F	0.4	2.9/49	Taussig Bing anomaly	–	NI	No	1.6	93	BiV	BAS
9	F	2.1	3.4/50	VSD, PDA, ASD	T21	NI	No	3	100	BiV	–
10	F	2.4	2.2/43	Multiple VSD, PDA	T18	I	Yes	3	97	Palliative	–
11	F	2.1	1.9/42	VSD, PDA	T13	I	Yes	3.4	100	Palliative	–
12	F	1.1	2.4/49	AVSD	T21	NI	No	4	100	BiV	–
13	F	0.9	3.9/53	Complete AVSD, PDA s/p PDA ligation	T21	I	Yes	4	100	BiV	–
14	F	0.2	2.8/50	HLHS (MA, AA)	–	I	Yes	N/A	96	UiV	Ductal stenting, BAS

AA, aortic atresia; AS, aortic stenosis; ASD, atrial septal defect; AVSD, atrioventricular septal defect; BAS, balloon atrial septostomy; BiV, biventricular physiology; CHD, congenital heart disease; CN, case number; CoA, coarctation; HLHS, hypoplastic left heart syndrome; I, invasive; LV, left ventricle; LVOTO, left ventricular outflow tract obstruction; MA, mitral atresia; N/A, non-available; NI, non-invasive; PA, pulmonary artery; PDA, patent ductus arteriosus; PFR, pulmonary flow restrictor; PV, pulmonary valve; RV, right ventricle; S/P, status post; T, trisomy; UiV, univentricular physiology; VSD, ventricular septal defect; W/H, weight (Kg)/height (cm).

^a^ Reason for delayed intervention.

### 3.1. Procedure

At the time of the intervention, 7 patients were intubated, 7 had non-invasive ventilation support and 6 had inotropic support. Three patients had balloon atrial septostomy. No branch PA stenosis was identified on baseline angiography. The median diameter of LPA was 4.8 mm (IQR, 4.1–6.1) at the proximal segment and 4.5 mm (IQR, 3.7–5.8) at the distal segment. The median diameter of RPA was 5.4 mm (IQR, 4.6–6.5) at the proximal segment and 5.1 mm (IQR, 4–6.1) at the distal segment. Two MVP-5Qs were wasted. In patient no. 5, MVP-5Q was pulled out, after release, with a 7 mm micro-snare from the LPA because it was loose and was upsized to MVP-7Q. In patient no. 8, we retrieved one MVP-7Q from the LPA before release because the patient became cyanotic. We tried the MVP-5Q but it was loose and was removed. We re-implanted the previously used MVP-7Q after adding another triangle. The Doppler pattern was not the reason to switch devices in both patients.

We obtained a systolic-diastolic Doppler flow profile in all patients before device release. It is noteworthy that we haven't witnessed hemodynamic changes after first implanting the left PFR. At the end of the intervention, the baseline Qp/Qs significantly decreased from a median of 3 (IQR, 2.5–3.5) to a median of 1.4 (IQR, 1.1–1.6) (*p* < 0.001). The baseline oxygen saturation significantly decreased from a median of 100% (IQR, 96.7%–100%) to a median of 85% (IQR, 82%–88.3%) (*p* < 0.001). Four patients had subsequent ductal stenting. The median overall fluoroscopy time was 27.5 min (IQR, 20.7–37.4). In patient no. 5, short-run supraventricular tachycardia was treated with cold saline infusion. There was no device embolization or procedure-related death. The procedural data and clinical outcomes are outlined in Table 4.

**Table 4:** Procedural data and clinical outcomes.

CN	Access	Prox/dis LPA Ø	Left MVP size	Left PFR Fenestration	Prox/dis RPA Ø	Right MVP size	Right PFR Fenestration	FT	Qp/Qs^a^	Spo2^a^	Delay to PFR Explantation (months)/Technique	FU	Status
1	RFV/5Fr	8.3/7.2	9Q	2 triangles	7.8/7.5	9Q	2 triangles	29	1.2	88	16.8/Forceps	19.5	Alive/BiV
2	RFV/6Fr	5.2/5	7Q	2 triangles	5.6/5.2	7Q	2 triangles	37.2	1.4	80	6/Forceps	15.2	Alive/BiV
3	RFV/4Fr	4.3/4.1	5Q	2 triangles	5.2/5.1	7Q	2 triangles	38.3	1.6	84	5.2/Forceps	13.1	Alive/BiV
4	RFV/5Fr	6.5/6	7Q	1 triangle	6.8/6.5	7Q	1 triangle	15	1.9	89	–	6.8	Dead, Seizures, Respiratory arrest
5	RIJV/6Fr	4.1/4.3	7Q	1 triangle	4.2/4.1	7Q	1 triangle	24.1	1.2	85	–	8.4	Alive/Aiming BiV
6	RFV/5Fr	7/6.7	9Q	1 triangle	6.5/6	9Q	1 triangle	25.2	1.7	90	1.5/Snare	8.3	Alive/BiV
7	RFV/5Fr	6/5.7	9Q	1 triangle	6.6/6.5	9Q	1 triangle	17	1.7	92	4.3/Forceps	6.4	Alive/BiV
8	RFV/6Fr	4.7/3.5	5Q	2 triangles	5/3.7	7Q	2 triangles	35	1.1	80	2/Forceps	2.6	Alive/BiV
9	RFV/6Fr	5.5/5.2	9Q	1 triangle	5.8/5.5	9Q	1 triangle	29	1.2	85	–	2.4	Alive/Aiming BiV
10	LFV/4Fr	5/4.7	7Q	1 triangle	6/5.8	9Q	1 triangle	22	1.6	82	–	1	Dead, Respiratory arrest
11	LFV/4Fr	4/3.8	5Q	2 triangles	4.3/4	5Q	2 triangles	38	1.6	87	–	2.7	Alive/Palliative
12	RFV/4Fr	3.5/3.2	5Q	2 triangles	4/3.8	5Q	2 triangles	16	1	85	–	2	Alive/Aiming BiV
13	RFV/4Fr	4.6/4.2	7Q	2 triangles	4.7/4.5	5Q	2 triangles	43	1	82	–	2	Alive/Aiming BiV
14	RFV/4Fr	3.9/3.4	5Q	1 triangle	4.7/3.9	7Q	1 triangle	26.1	–	83	1.2/Forceps	4.2	Dead, Post-Glenn severe NEC-associated sepsis, Care withdrawn

BiV, biventricular physiology; CN, case number; FT, fluoroscopy time (min); FU, follow-up (months); LFV, left femoral vein; LPA, left pulmonary artery; NEC, necrotizing enterocolitis PFR, pulmonary flow restrictor; Prox/dis, proximal/distal; RPA, right pulmonary artery; MVP, microvascular plug; RFV, right femoral vein; RIJV, right internal jugular vein; Ø, diameter (mm).

^a^ At the end of the procedure.

### 3.2. Post-procedure care

There were no immediate postoperative management challenges. Extubation was achieved after a median 1 day (IQR, 1–2) postoperative. No inotropic support was needed after extubation. All patients were discharged from the intensive care unit after a median of 3.5 days (IQR, 2–5.8) postoperative. At hospital discharge, the median non-invasive oxygen saturation was 90% (IQR, 88%–91%), the median maximum velocity of the continuous-wave Doppler tracing was 3.9 m/s (IQR, 3.3–4.5) on LPA and 3.9 m/s (IQR, 3.2–4.7) on RPA.

### 3.3. Follow-up

**Figure 3:**
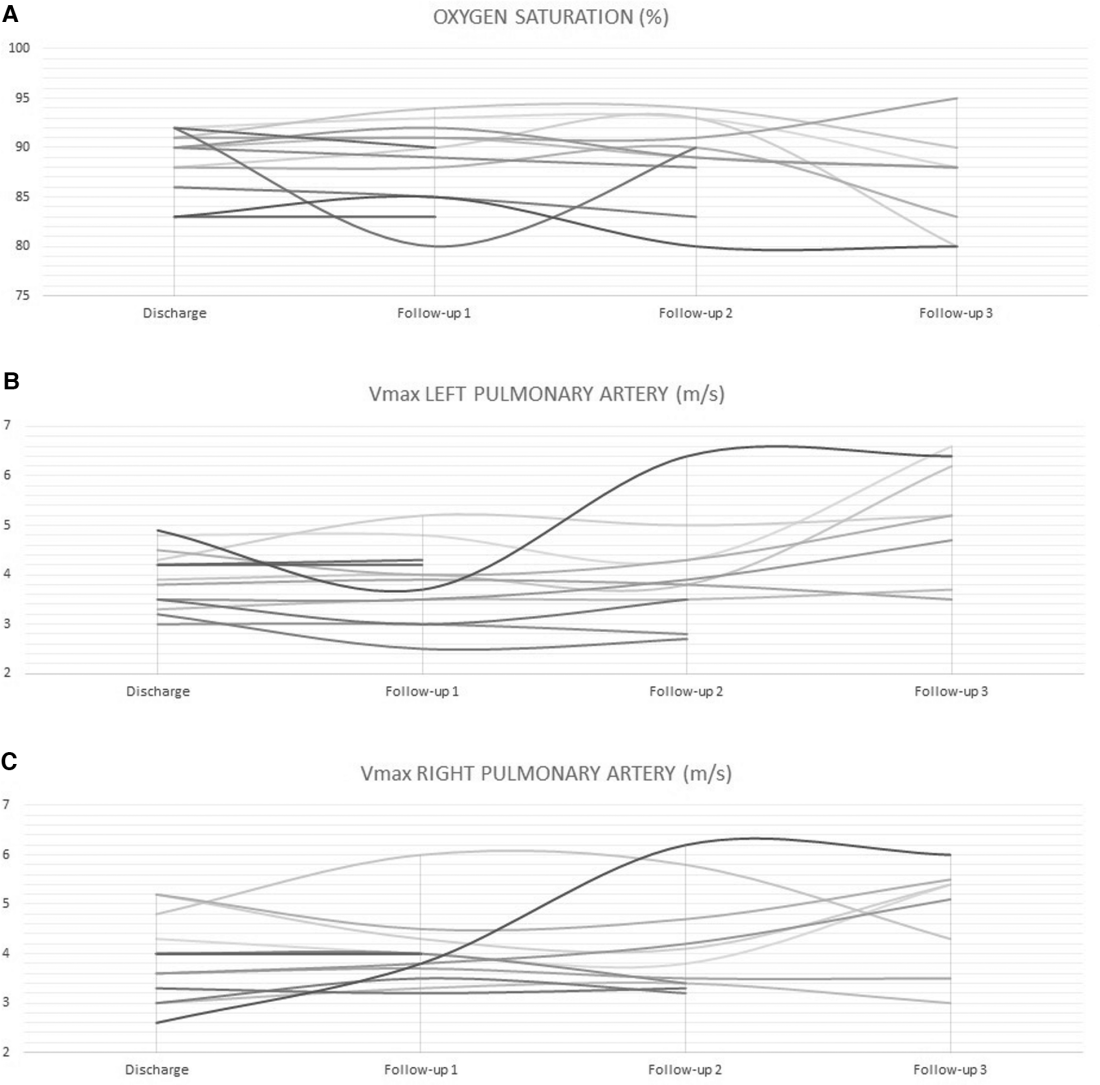
Limited variability in oxygen saturation (**A**) and maximum velocity of the continuous-wave Doppler tracing on LPA (**B**) and RPA (**C**) during follow-up.

**Figure 4:**
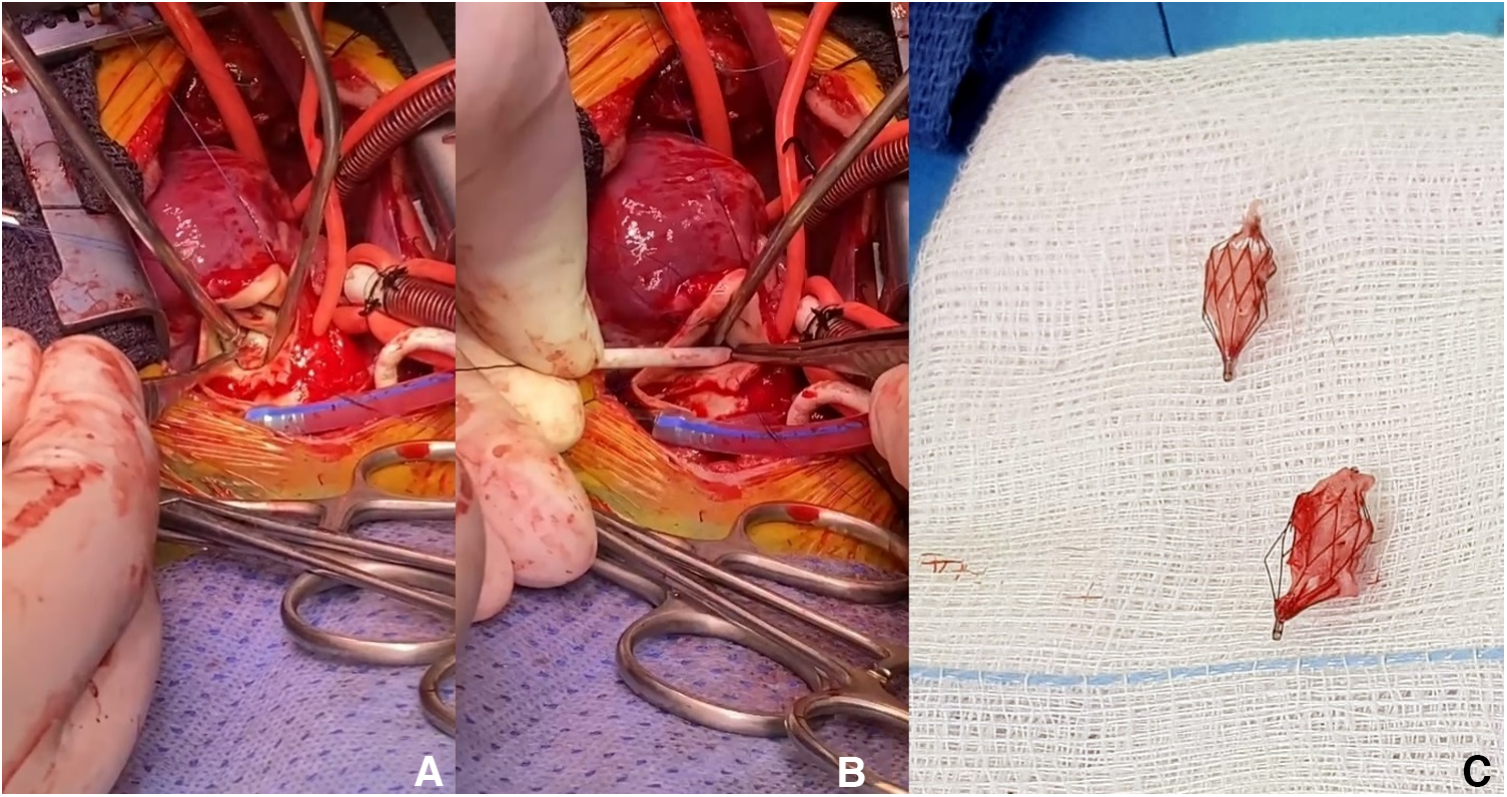
Intraoperative snare-removed PFRs in patient no. 6 (A, B, and C).

**Figure 5:**
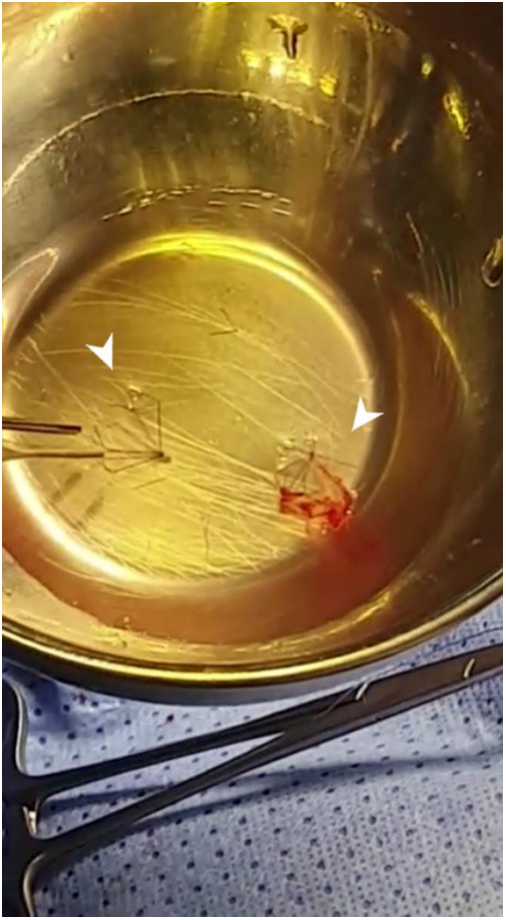
Fragile MVP-based PFRs removed in pieces (white pointed arrows) from patient no. 1.

On a median follow-up of 5.3 months (IQR, 2.4–8.4), there was limited variability in the oxygen saturation and the maximum velocity of the continuous-wave Doppler tracing on both PA branches (Figure 3). Five patients (no. 1, 2, 3, 5, and 14) had routine inter-stage catheterization which showed normal end-diastolic ventricular pressures, no PFRs migration or branch PA stenosis or distortion. We also did not observe any filling defect on control angiograms. Of these five patients, redo catheterization in patients no. 3 and 5 with borderline left ventricle demonstrated adequacy for bi-ventricular circulation. Fourteen (50%) PFRs were surgically explanted uneventfully on a median of 4.3 months (IQR, 1.2–6) post-implantation during biventricular repair in six patients and stage-2 palliation in patient no. 14. The removal of the MVP was done under direct vision using forceps in six patients (Supplementary Video S6) and a snare catheter in one patient (no. 6) through a longitudinal opening on the anterior surface of the MPA (Figure 4). We did not observe any thrombus formation on the removed devices. In patient no.1, we first attempted snare removal but it was not possible to retrieve the device inside the sheath. The device was adherent to the vessel wall and covered with neo-endothelium. We opened the branch PAs directly over the devices which were separated from the wall by dissection. Both fragile PFRs were removed in pieces (Figure 5) and both branch PAs were repaired with autologous pericardium. In patient no. 14, the left PFR was removed easily leaving a widely patent vessel. The right PFR was removed piecemeal as it was partially adherent to the vessel wall with some intimal laceration anteriorly needing repair with a small autologous pericardial patch. The patency and size of the branches were confirmed adequate by appropriate Heggar dilators. There was no need for an exit angiography or redo catheterization in the six patients with biventricular hearts after repair, and all of them had an uneventful follow-up. The atrioventricular valve regurgitation in three patients with AVSDs did not get worse after the procedure. Four other patients are scheduled for surgical PFR removal and biventricular repair. One patient (no. 11) with trisomy 13 is alive at 2.7 months post-procedure. There were three late deaths. Two patients (no. 4 and no. 10) with trisomy 18 died at 1 and 6.8 months after the procedure from non-cardiac causes. Patient (no. 14) had stage-2 palliation 3.2 months after PFRs implantation. He died 1 month after surgery from severe necrotizing enterocolitis-associated sepsis. He had a post-Glenn angiogram which showed no branch PA stenosis.

## 4. Discussion

Conventional surgical PA banding has been practiced for almost 70 years, but it is a procedure that carries multiple potential risks and morbidities (6, 7). This palliative surgery aimed to control excessive pulmonary blood flow in an era when corrective surgery for neonates was too risky or not available. Banding the PA has recently gained interest for left ventricular retraining and hypoplastic left heart malformations (3). Transcutaneously adjustable, dilatable, and resorbable PA bands like the Flo Watch have been trialed without widespread use in standard clinical practice (8, 9). Attempts have been also made to develop a transcatheter, implantable PFR without great success (10, 11). The recent development and successful use of MVP-based bespoke PFRs in animals and humans was an important turning point in the field of transcatheter interventions, making great strides in completing the Norwood stage-1, totally percutaneously (1, 2, 4, 12). We thought and showed that this appealing prospect can be safely and effectively applied to any lesion in which balanced pulmonary and systemic circulation is needed.

We observed that all patients with intra-cardiac shunts did well, especially when treated early. The vascular pulsatility is more apparent in QP/QS mismatch lesions and the PA cross-sectional shape is not completely circular both leading to higher odds of para-device leaks. For those reasons, early interventions when PA branches are not yet dilated and over-pulsatile should give better results when compared to later interventions beyond 6 weeks of age. Having a large ductal shunt on top of the intracardiac shunt will increase the oxygen saturation but the symptoms will improve. This is not surprising because the arterial duct will shunt in the systolic and diastolic phases while VSD will shunt only in the systolic phase.

Post-natal biventricular repair is uncertain in patients with a small ventricle. The hypoplastic ventricle must be rehabilitated using a balanced procedure that allows adequate blood flow in the ventricular cavity for further post-natal growth of the borderline left heart structures. This new appealing transcatheter concept turned out also a safe and effective strategic choice for patients with borderline left heart structures to avoid sophisticated neonatal biventricular repair and to lay the basis for further post-natal growth of the sub-aortic chambers (13).

### 4.1. Technical challenges and risks

Modified MVP is an attractive device to be used as an endovascular band due to its low profile, smooth delivery, easy positioning, and retrievability (14). However, there are some technical challenges and risks to overcome. Fenestrating the MVP with a scalpel has been reported to be difficult and the ability to ascertain the appropriately sized fenestration is the biggest challenge. Nageotte et al. reported that patients continued to demonstrate evidence of pulmonary over-circulation with elevated distal PA pressures despite appropriate device sizing as confirmed on angiography and overflow restriction as confirmed on ultrasound (4). They attributed some of that to para-device leaks. Another reason for continuous pulmonary over-circulation may be because the restriction occurs only at the level of a very thin PTFE membrane which may not impose enough resistance to blood flow when subjected to systemic pressures. Bespoke MVPs are placed into a high-pressure, high-flow circulation. This means that the ratio of the device to the measured lumen of branch PA should be chosen generously to the larger MVP size, as it was previously recommended by Haddad and colleagues in their large series on MVP-based vascular embolization (14). This is an important point to consider because previous authors also raised concerns about the slightly tangential orientation of the undersized device in tortuous vessel walls as well as the higher risk of device distal migration with undersized diameters and high flow circulations (15, 16). We implanted the MVP-based PFRs in 1 mm smaller PAs than the team of Schranz in their pioneering human series (2). One of the major challenges over here is to comprehensively balance the oversizing in a way to ensure stable implantation of the device while making sure not to excessively oversize these devices because the PTFE covering of the plug needs to open fully, otherwise, a para-device leak will occur, making the PFR not ideal (17).

Customization of the MVP is a new highly appealing concept and the overall experience is limited to two reports (2, 4). Every novel technique and experience needs a “word of caution” and that's why both of these experiences with their learning effects complement each other (2–4). Ballooning of the fine, very thin PTFE membrane as described by Nageotte et al. is questionable as is crossing the just created PFR by catheters just for unnecessary pressure readings. They created the fenestration with a low-temperature fine-tip Eye Bovie cautery and dilated it with a 3 mm coronary balloon (4). Some of the hybrid literature suggests making PA bands closer to 2.5 mm in luminal diameter (18). Perhaps that should have been the ideal fenestration size to aim for in small HLHS patients, as Nageotte et al. discussed in their report (4). Based on our experience, we achieved clinically effective PFRs by fenestrating one triangle on the MVP-7Q and MVP-9Q as well as two triangles in two diamonds on the MVP-5Q. These fenestrations are, in optimal *ex-vivo* conditions, equivalent to the overall surface area of a circle PA band with an inner diameter of 2.8, 3.9, and 4.4 mm in luminal diameter, respectively (Table 2). In retrospect, we think that the 2 fenestrations on the MVP-5Qs were generous but we were not confident of leaving patients with only one triangle. In practice, we did not take these calculated diameters in accordance and the fenestrations were tailored patient-to-patient, device-to-device as described earlier in the methods. In view of the fact that oversized MVPs were also used here for stable positioning of the PFRs within the PA branches, it remains to be seen whether the openings created in the device remain unaffected *in-vivo*.

We used the multipurpose GlideCath and did not replace it with a slightly angled Judkins right or Cobra-shaped glide catheter for PFR placement as has been previously reported by other colleagues (2–4). This approach might be questionable, particularly for the in-part 90° angled RPA entrance. We deployed the MVP device by rapid unsheathing (catheter withdrawal) rather than positioning the catheter more proximally in the PA branch and pushing the device forward into the landing zone. With this approach, we observed that the end shape of the catheter did not have a major effect on having a stable catheter position and controlled implantation (Supplementary Video S3). We didn't deal with high tension on the delivery cable, or even dislodgement and the need for repositioning while attempting to place the MVPs. In fact, the MVP device is extremely light and soft and there was almost no tension on the delivery cable. Adopting the unsheathing technique for device implantation was straightforward in all cases.

We acknowledge the risk of jailing the upper lobe branches. However, this risk is minimized with appropriate device size to ensure stable positioning, keeping in mind that MVP distal part is not covered. The soft Nitinol skeleton allows to retract and re-position the MVP until satisfactory implantation is obtained. Hence, care should be taken with repeated device re-sheathing as this may lead to small tears in the PTFE membrane (19). It has been reported that these devices tend to migrate distally, especially in the RPA which can potentially jail the distal branches and subject the upper lobe arteries to high pressure (4). The RPA is usually larger and longer but the right upper PA lobe branch can sometimes take off from a more proximal area. The RPA is also closer to the pulmonary valve and the MVP elongation can get close to the pulmonary valve. Smaller/shorter MVPs could be a better option in some cases. Invasive pressure measurement with a catheter retraction technique through the PFR is useless and risky. The effectiveness of the PFR can be demonstrated by ultrasound (Figure 2).

### 4.2. Benefits

By uncovering a specific number of triangles at the proximal end, one could accurately predict the theoretical *ex-vivo* luminal size of the PA band and control the degree of pulmonary flow restriction (Table 2). The MVP customization can be performed quickly on the table. Heparinization for the procedure and anti-platelet medications may be required to maintain luminal patency in the long run. We have not seen any thrombosis-related complications. However, we didn't feel confident in leaving the patients on a single antiplatelet agent (3). This is something that we will probably move to shortly.

Retrieval of those plugs is technically easy and it has been previously described (1–4). Although we didn't have to snare any plug on another occasion that the deployment procedure, we believe that snaring the plug within the first 3 weeks will be easy without damaging the vessel (2). Even though Khan et al. reported that 50% of MVP devices can be removed by snares after 12 weeks (1), snaring out these devices 4 weeks after implantation appears hazardous, leading to PA damage and narrowing. Our surgeons described their experience in device explantation. We feel more confident that those plugs were easy to explant, either by pulling them out, under direct view, either in one piece or in pieces. During removal, it is important to secure the proximal end of the MVP with forceps. The proximal half contains the PTFE membrane and can be easily separated away from the distal half and the vessel wall. It is essential to pull out the covered part in one piece, which was the case in our series (Figure 5). The remaining bare part of the device can be embedded in the endothelium and pieces could be removed one by one. The other interesting finding was using the snare technique in the operating theatre to explant old PFRs, and also without the need to patch the vessel (Figure 4).

### 4.3. Limitations

Most of our patients had a weight higher than 2.5 kg and probably would not have major contraindications for surgical PA banding. We adopted this promising novel transcatheter deliverable endoluminal PA bands to avoid one surgical step and sternotomy. There is a learning curve to achieve before taking this practice to a wider scale. Longer-term comparison studies are necessary to demonstrate the benefits over existing therapies. Treating PA branches with diameters beyond 8 mm will likely cause distal migration of the MVPs exposing the upper lobe branches or jailing them. More importantly, the para-device leak is more considerable when reaching those sizes.

We adopted the usual practice of monitoring the efficacy of surgical PA bands, which is the peak Doppler gradient. Previous teams noticed that the diastolic gradient increases over time resulting in increases in the mean gradient. We, however, did not record the mean gradients to discuss that observation. It is also noteworthy that hemodynamics including PFR characteristics are different in anesthetized or deeply sedated patients and the PFR effects can be very different. Effectiveness of the endoluminal PA band determined by clinical signs and Doppler pattern should be also analyzed in terms of cardiovascular co-medications affecting residual or intermittent overflow conditions. Speculating about the definition of an “optimal” sized hole for a PFR is highly debatable. For those reasons, we used several parameters to evaluate the efficacy of the endoluminal band such as oxygen saturation, blood pressure, angiograms, cardiac index, and ultrasound peak systolic gradients.

Our experience with the percutaneous stage-1 approach in patients with HLHS is limited to one. During surgical PFR removal in patient no.14, the left side was unproblematic, but there was a laceration on the right side. Patch expansion of the LPA or prophylactic stent placement can be needed during stage-2, but should not be necessary (3). However, problem on the RPA are not usually expected. Considering that the Glenn anastomosis is usually performed at the side of RPA, such laceration could have been solved by the surgical connection of the superior vena cava to the RPA. However, in patient no. 14, our surgeon judged that a small patch was needed for a more esthetical surgical outcome. This is a point perhaps important in view of future HLHS treatments using the minimally invasive transcatheter method as stage-1 palliation routinely.

We had a small group of patients with fatal trisomy disorders who were stuck on the mechanical ventilator for multiple reasons, including the left-to-right shunt. Although we managed the QP/QS mismatch, as demonstrated by invasive measurements, these patients are not always going to benefit from the intervention. They have more complex respiratory, neurological, and muscular problems related to the syndrome itself that could prevent the child from benefiting from the procedure or lead to death.

### 4.4. Future perspectives

We applaud the pioneering efforts of Schranz and colleagues in promoting this novel technique in percutaneous stage 1 palliation. We expand upon their findings and report the safety and efficacy of this concept in various congenital heart lesions. As we described earlier in detail, the shape and dimensions of the MVP device are not ideal. The distal part of the MVP device is not covered, and this helps to avoid pinching or jailing the upper lobe PA branches. On the other hand, the surgical perspective could be that the uncovered part of the device is responsible to grow too much into the vessel wall. Based on your initial experiences, we think that there is room in the industry to develop ready-to-use PFRs with perfectly created fenestrations. A custom-made device for transcatheter endoluminal PA banding should be first deliverable through a 4-Fr catheter and has a distal marker for accurate positioning together with a proximal radiopaque micro pin for mechanical delivery and easy snare-recapture of the device, if necessary. In comparison with the MVP, the custom-made device should be shorter with 8 and 10 mm lengths which both are sufficient for a stable landing. It should also be designed in 5 diameters starting from 5 mm and incrementally moving in 1.5 mm up to 11 mm for a more tailored approach while respecting a reasonable 1.5 mm of additional oversizing. The proximal parachute covering should include 80%–100% of the device length as we think this is necessary to obtain a stable PTFE membrane while reducing the length of the bare-metal device part. A double-layer sandwich PTFE covering design could be beneficial to obtain a thicker PTFE membrane ready to impose enough resistance to blood flow when subjected to high-pressure, high-flow circulation. The fenestration should be either circular or oval-shaped, centrally created, shallow, and most importantly metal-reinforced to obtain an accurate *in-vivo* size of the fenestration. In our opinion, the ideal diameters of the fenestrations could be 2.8, 3.2, and 3.6 mm.

## 5. Conclusion

In our experience, we showed that MVP-based bespoke PFRs can be effectively used as short-term palliation in two ventricle patients with excessive pulmonary blood flow before complete repair. Patients with a single ventricle program or with borderline physiology are also good candidates for this appealing technique but are more challenging cases because perfectly balanced pulmonary and systemic circulation is critical to their future success. Surgical explantation during subsequent palliative or definitive surgeries is fairly straightforward with no residual vessel injury noted.

## Data Availability

The raw data supporting the conclusions of this article will be made available by the authors, upon request, to any qualified researcher.

## Supplementary material

Supplementary Video S1 Baseline angiographies in 30° left (left panel) and right (right panel) anterior oblique planes for accurate measurements of PA branches.

Supplementary Video S2 Patient no. 12. Fluoroscopic view in 30° left anterior oblique (left panel) and lateral 90° (right panel) showing how the 4-Fr multipurpose GlideCath is railed over the telescoping microcatheter system and placed in the distal LPA.

Supplementary Video S3 Patient no. 12. Fluoroscopic view in 30° left anterior oblique (left panel) and lateral 90° (right panel) of right PFR implantation through a 4-Fr multipurpose GlideCath.

Supplementary Video S4 Patient no. 12. Completion angiographies in cranially tilted anteroposterior (left panel) and lateral 90° (right panel) planes after placement of modified MVPs within the PA branches.

Supplementary Video S5 Patient no. 8. Completion angiographies in cranially tilted anteroposterior (left panel) and lateral 90° (right panel) planes after placement of modified MVPs within the PA branches.

Supplementary Video S6 Patient no. 8. Intraoperative smooth removal of both PFRs with the dissector technique.
